# Women’s healthcare decision-making and unmet need for contraception in Mali

**DOI:** 10.1186/s12978-022-01484-w

**Published:** 2022-08-20

**Authors:** Edward Kwabena Ameyaw, Abdul-Aziz Seidu, Bright Opoku Ahinkorah

**Affiliations:** 1 School of Public Health, Faculty of Health, University of Technology Sydney, Sydney, NSW Australia; 2 Centre for Gender and Advocacy, Takoradi Technical University, Takoradi, Ghana; 3 College of Public Health, Medical and Veterinary Sciences, James Cook University, Townsville, QLD 4811 Australia

**Keywords:** Unmet need, Contraception, Family planning, Mali, Reproductive health

## Abstract

**Background:**

Contraception plays a significant role in fertility regulation. Evidence suggests that reproductive health rights influence contraception use. Women of Mali are noted to have limited control over their healthcare decisions. As a result, this study aimed at investigating the association between women’s healthcare decision-making capacity and unmet need for contraception in Mali.

**Methods:**

This study comprised 6593 women who participated in the 2018 Mali Demographic and Health Survey. Two binary logistic regression models were built. Whilst the first model (crude) involved healthcare decision-making capacity and unmet need for contraception, the second one was a complete model which controlled for all the socio-demographic characteristics. Sample weight was applied and Stata version 13.0 was used for all analyses.

**Results:**

Most of the women were not taking their healthcare decisions alone (92.8%). Nearly four out of ten of them indicated that they had unmet need for contraception (35.7%). Unmet need for contraception was high among women aged 45–49 (50.9%) and low among those aged 15–19 (19.2%). Unmet need for contraception was more probable among women who took their healthcare decisions alone compared to those who did not take their healthcare decisions alone [AOR = 1.35; CI = 1.08–1.70]. Compared with women aged 15–19, unmet need was higher among women aged 45–49 [AOR = 4.58, CI = 3.05–6.86]. Richer women had lower odds of unmet need for contraception compared with poorest women [AOR = 0.77, CI = 0.61–0.97].

**Conclusion:**

Women who took their healthcare decisions alone had higher odds of unmet need for contraception. To increase contraceptive use in Mali, it is imperative to take women’s healthcare decisions into consideration to strengthen existing policies geared towards fertility control and improvement in maternal health to achieve Sustainable Development Goals 3 and 5. Sustainable Development Goal 3 seeks to ensure healthy lives and promote well-being for all at all ages whilst Goal 5 aims at achieving gender equality and empower all women and girls.

## Plain Language summary

Evidence suggests that reproductive health rights influence contraception use. In Mali, women are noted to have limited control over their healthcare decisions. Due to that, this current study explored the relationship between women’s healthcare decision-making capacity and unmet need for contraception in Mali. The sample was 6593 women aged 15–49. About nine out of ten of the women were not taking their healthcare decisions alone and 35.7% indicated that they had unmet need for contraception. Unmet need for contraception dominated among those aged 45–49 (50.9%) but was low among those aged 15–19 (19.2%). Women who took their healthcare decisions alone had high odds of unmet need for contraception. Compared with women aged 15–19, unmet need was nearly five times likely among women aged 45–49. Richer women had lower odds of unmet need for contraception compared with poorest women. To increase contraceptive use in Mali, it is important to take into consideration the variations revealed in this study. This will help to strengthen existing policies geared towards fertility control and improvement in maternal health.

## Background

Childbearing has been considered an essential event in the life cycle of a woman, especially across the span of her reproductive life [1]. Pregnancy and childbirth often comes with life-changing experiences both in physical and psychosocial dimensions and are mostly associated with intense emotional rewards as well as strains [2, 3]. Due to the intense physical and emotional strains that can occur during this period in a woman’s life, women are encouraged to take necessary preparations (taking healthy diet, managing stress, controlling body weight and finances) to safeguard themselves from adverse birth outcomes such as preterm births and low birth weight [4, 5]. Despite these expectations, globally, a lot of women remain unaware of the advantages of a planned pregnancy [6], and often end up with pregnancies that are unintended, mostly occurring in sub-Saharan Africa [7, 8]. Some of the major reasons behind unintended pregnancy are the lack of proper family planning methods and unclear fertility goals [1].

Globally, it has been recognised that the use of contraceptives plays a significant role in regulating fertility [9]. This is a key aspect of the reproductive health of women who wish to space or limit childbearing [10]. A useful measure of the gap between the reproductive desires of women and access to contraception is the estimation of unmet need for contraception [11]. The World Health Organisation (WHO) describes women with unmet need for contraception as those who are fecund and sexually active but are not using any method of contraception, and report not wanting any more children or wanting to delay the next child [12]. Other scholars also refer to such women as women who wish to space or limit births but do not use contraceptive methods to achieve this [13–15]. This indicator is essential not only because it informs and guides contraceptive services, but also indicates a country’s compliance with the reproductive health rights of its population [10].

Despite the link between access to contraception and reproductive health rights, women of Mali have been found to lack reproductive self-determination reinforced by lack of access to family planning methods [16]. Coupled with this, the law in Mali and medical providers further undermine women’s autonomy by demanding that women seek authorization for certain contraceptive procedures from husbands and requires minors to obtain parental authorization for family planning services [16]. Women’s ability to seek care in the first place is impeded by their lack of decision-making power within their families in Mali [16]. All these situations explain why the country recorded contraceptive prevalence of 17% (16% modern and 1% traditional) in 2018 [17]. In spite of this, there has not been any study that has attempted to establish a link between healthcare decision-making capacity and unmet need for contraception in Mali. It is, however, essential to understand whether there is a link between the capacity of women in Mali to make decisions on their health and unmet need for contraception. This study, therefore, seeks to fill this gap by examining the role of women’s healthcare decision-making capacity on unmet need for contraception in Mali using data from the 2018 Mali Demographic and Health Survey (MDHS). We hypothesized that women who take decisions alone on their healthcare are more likely to have no unmet need for contraception.

## Methods

### Data

The data used for this study forms part of the 2018 MDHS. The women’s recode file was used in this current study. The survey is the sixth version since its commencement in 1987 [17]. The MDHS is part of the DHS Program which aims at monitoring health indicators in low and middle-income countries. The aim of the survey is to offer reliable estimates of family planning methods, fertility preferences, sexual activity, marriage and other essential population health measures. It utilised a two-stage sampling design. At the primary stage, 379 Primary Survey Units (PSU)/clusters from urban (104) and rural (275) areas were systematically selected [17]. These were selected through probability proportional to the size of households. This was done from the list of Enumeration Sections (ES) which were established during the General Census of Population and Housing (GCPH) conducted in 2009.

Household mapping and enumeration operation in the clusters were executed in order to obtain an updated list of households in each ES. Mapping and enumeration of households were conducted between May 25 and July 8, 2018 within the regions of the country. However, in the case of Kidal, Gao and Timbuktu regions, mapping and enumeration were done shortly before the main survey. After this stage, 35 households were sampled from Kidal, Gao and Timbuktu regions through a systematic draw having equal probability. The same procedure was followed to select 26 households from all the other regions. All women between 15 and 49 years who lived in the selected households or were present the night before the survey were considered eligible to participate in the survey. This resulted in a nationally representative sample of 10,519 women. The survey had 98.0% response rate for the women [17].

### Sample and inclusion criteria

A total of 6593 women were included in the current study. These are women who responded to the dependent variable (unmet need for contraception) and the key independent variable during the survey, thus healthcare decision-making capacity. This notwithstanding, we excluded those without complete data on the selected socio-demographic characteristics.

### Derivation of variables

#### Outcome variable

The outcome variable for this study was unmet need for contraception, that is whether women had unmet need for contraception or not (i.e. yes = 1/no = 0). It was derived by aggregating unmet need for limiting and spacing. A married or cohabiting woman had unmet need if she did not want any further child or want to delay her next birth for at least 2 years but not making use of contraception. Women who were pregnant or amenorrheic and had unwanted or mistimed births or pregnancies were also considered as having unmet need if such women were not using contraception when they got pregnant [18–20].

#### Independent variable

Healthcare decision-making capacity was the principal independent variable for the study. It was derived from the question “Who usually makes decisions about health care for yourself: you, your (husband/partner), you and your (husband/partner) jointly, or someone else?” The following were the responses to the question; respondent, husband/partner, respondent and husband/partner jointly, someone else, other. These responses were coded as alone (respondent) = 1 and not alone (consisting of husband/partner, respondent and husband/partner jointly, someone else, other) = 0 as conceptualised by some previous studies [21, 22]. Women’s healthcare decision-making capacity was the key independent variable for the study because women in low and middle-income countries, including Mali, usually have limited autonomy and control as far as their health decisions are concerned [23]. This has been noted to influence their contraceptive use prospects [24, 25]. Eleven covariates were considered in addition; age, wealth status, marital status, religion, education, partner’s education, residence, frequency of reading newspaper/magazine, frequency of watching television, frequency of listening to radio, and occupation following their availability in the dataset and conclusions drawn by some previous studies conducted in Africa about their association with unmet need [25–27]. In order to make all these variables conceptually meaningful and suitable for the analysis, some of them were recoded. These are marital status coded as married = 0 and cohabiting = 1; religion coded as Islam = 0, Christianity = 1, Traditional religion = 2 and no religion = 3; and lastly occupation coded as not working = 0, managerial = 1, clerical = 2, sales = 3, agriculture = 4, services = 5, manual = 6.

### Statistical analyses

We employed descriptive and inferential analytical techniques in assessing the influence of healthcare decision-making capacity on unmet need for contraception. The descriptive analysis involved computation of the healthcare decision making capacity of the study participants. Their socio-demographic characteristics were also calculated. This was followed by bivariate analysis of healthcare decision-making capacity and unmet need for contraception, where associations were explored with chi-square statistics and p-values. The associations were considered significant at 95% level of significance as shown in Table 1. The inherent sample weight was applied at this stage in order to mitigate deficiencies such as under or over representation which usually compromise the quality of surveys of this kind [28].

**Table 1:** Healthcare decision making capacity, socio-demographic variables and unmet need for contraception (n = 6,593)

Variable (p-value)	Weighted frequency (n)	Weighted percentage (%)	Unmet need	
			Yes (%)	No (%)
Healthcare decision making capacity (χ^2^ = 17.1; p < 0.001)				
Not alone	6121	92.8	26.6	73.4
Alone	472	7.2	35.7	64.3
Age (χ^2^ = 184.9; p < 0.001)				
15–19	764	11.6	19.2	80.8
20–24	1347	20.4	22.8	77.2
25–29	1589	24.1	23.1	76.9
30–34	1271	19.3	28.4	71.6
35–39	978	14.8	33.8	66.2
40–44	477	7.3	43.7	56.3
45–49	167	2.5	50.9	49.1
Wealth Quintile (χ^2^ = 44.8; p < 0.001)				
Poorest	1260	19.1	33.7	66.3
Poorer	1345	20.4	27.6	72.4
Middle	1397	21.2	26.3	73.7
Richer	1344	20.4	26.9	73.1
Richest	1247	18.9	22.0	78.0
Marital status (χ^2^ = 0.6; p = 0.447)				
Married	6528	99.0	27.2	72.8
Cohabiting	65	1.0	31.3	68.7
Religion (χ^2^ = 12.3; p < 0.01)				
Islam	6178	93.7	26.9	73.1
Christianity	179	2.7	26.2	73.8
Traditional religion	40	0.6	32.3	67.7
No religion	196	3.0	39.7	60.3
Education (χ^2^ = 52.9; p < 0.001)				
No education	4702	71.3	29.3	70.7
Primary	799	12.1	26.8	73.2
Secondary	987	15.0	18.9	81.1
Higher	105	1.6	14.4	85.6
Partner’s education (χ^2^ = 42.9; p < 0.001)				
No education	4788	72.6	29.3	70.7
Primary	637	9.7	24.1	75.9
Secondary	863	13.1	21.5	78.5
Higher	305	4.6	17.3	82.7
Residence (χ^2^ = 0.1; p = 0.815)				
Rural	5189	78.7	27.2	72.8
Urban	1404	21.3	27.4	72.6
Frequency of reading newspaper/magazine (χ^2^ = 21.8; p < 0.001)				
Not at all	6219	94.3	27.8	72.2
Less than once a week	221	3.4	21.4	78.6
At least once a week	153	2.3	13.0	87.0
Frequency of watching television (χ^2^ = 13.0; p < 0.01)				
Not at all	2536	38.4	29.1	70.9
Less than once a week	1414	21.5	28.1	71.9
At least once a week	2643	40.1	24.8	75.2
Frequency of listening to radio (χ^2^ = 4.0; p = 0.135)				
Not at all	2051	31.1	28.8	71.2
Less than once a week	1505	22.8	27.1	72.9
At least once a week	3037	46.1	26.3	73.7
Occupation (χ^2^ = 85.2; p < 0.001)				
Not working	2624	39.8	25.2	74.8
Managerial	135	2.1	10.6	89.4
Clerical	15	0.2	18.8	81.2
Sales	1583	24.0	27.3	72.7
Agriculture	1850	28.1	35.3	64.7
Services	343	5.2	19.5	80.5
Manual	43	0.6	20.9	79.1

Source: 2018 Mali Demographic and Health Survey

Binary Logistic Regression analysis was conducted thereafter. As shown in Table 2, we built two models. In the first model, only the principal independent variable-healthcare decision -making capacity and unmet need for contraception were involved. Outcome of this model (Model 1) was presented as crude odds ratio (COR). In the second model, all socio-demographic variables that were found to be significant during the chi-square test were included. This has been reported as adjusted odds ratios (AOR) as shown in Table 2. We checked for the presence of multicollinearity among the variables fitted into the multivariate model (Model 2) with variance inflation factor (VIF). The VIF test indicated that no multicollinearity existed (maximum VIF = 2.8; mean VIF = 1.5). Additionally, the survey command (svy) in Stata was used in order to adjust for the complex sampling structure of the data. All analyses were carried out with Stata version 13.0.

**Table 2:** Binary Logistic Regression on healthcare decision making capacity, socio-demographic variables and unmet need for contraception

Variable	Model 1		Model 2	
	COR	95% CI	AOR	95% CI
Healthcare decision-making capacity				
Not alone	1	[1, 1]	1	[1, 1]
Alone	1.55***	[1.25–1.93]	1.35**	[1.08–1.70]
Age				
15–19			1	[1, 1]
20–24			1.19	[0.93–1.55]
25–29			1.21	[0.97–1.53]
30–34			1.70***	[1.36–2.15]
35–39			2.15***	[1.64–2.83]
40–44			3.20***	[2.37–4.31]
45–49			4.58***	[3.05–6.86]
Wealth				
Poorest			1	[1, 1]
Poorer			0.83	[0.68–1.01]
Middle			0.82*	[0.67–0.99]
Richer			0.77*	[0.61–0.97]
Richest			0.75	[0.56–1.01]
Religion				
Islam			1	[1, 1]
Christianity			0.99	[0.69–1.44]
Traditional religion			0.79	[0.30–2.09]
No religion			1.53**	[1.07–2.19]
Education				
No education			1	[1, 1]
Primary			1.05	[0.86–1.30]
Secondary			1.01	[0.80–1.30]
Higher			1.73	[0.38–1.39]
Partner’s education				
No education			1	[1, 1]
Primary			0.87	[0.68–1.11]
Secondary			0.85	[0.67–1.07]
Higher			0.84	[0.52–1.35]
Frequency of reading newspaper/magazine				
Not at all			1	[1, 1]
Less than once a week			1.01	[0.66–1.55]
At least once a week			0.58	[0.33–1.02]
Frequency of watching television				
Not at all			1	[1, 1]
Less than once a week			1.08	[0.89–1.30]
At least once a week			0.94	[0.78–1.13]
Occupation				
Not working			1	[1, 1]
Managerial			0.40**	[0.22–0.72]
Clerical			0.76	[0.23–2.48]
Sales			1.00	[0.86–1.17]
Agriculture			1.36***	[1.15–1.61]
Services			0.80	[0.51–1.25]
Manual			0.78	[0.33–1.89]

*p < 0.05, **p < 0.01, ***p < 0.001

Source: 2018 Mali Demographic and Health Survey

### Ethical approval

According to the 2018 MDHS report, ethical approval was granted by the Institutional Review Board of Inner City Fund (ICF) International [17]. Consent was also sought from each woman during the fieldwork. Informed consent was obtained from a parent or guardian for participants under 16 years old. The authors of this manuscript sought permission from the DHS Program for use of the dataset for this study. The data can be accessed from their website (www.measuredhs.com).

## Results

### Healthcare decision making capacity, socio-demographic variables and unmet need for contraception

Table 1 presents the results of the descriptive analysis. Most of the women were not taking their healthcare decisions alone (92.8%). Nearly four out of ten of them indicated that they had unmet need for contraception (35.7%). Unmet need for contraception was high among those aged 45–49 (50.9%) and low among those aged 15–19 (19.2%). Unmet need for contraception was relatively high among poorest women (33.7%) but was least reported by richest women (22.0%). A significant section of the cohabiting women had unmet need for contraception (39.7%). Unmet need for contraception was dominant among women who had no religion (39.7%), meanwhile, only 26.2% of the Christians had unmet need for contraception. Seven out of ten had no formal education (71.3%) and unmet need for contraception was relatively high among same (29.3%).

Unmet need stood at 29.3% among those whose partners had no formal education but was 17.3% for women whose partners had higher education. The prevalence of unmet need for contraception was almost the same for rural (27.0%) and urban (27.4%) residents. Most of the women did not read newspaper at all (94.3%) and unmet need was significantly reported by the same category of women (27.8%). Nearly three out of ten of those who did not watch television at all (29.1%) had unmet need for contraception. Unmet need was phenomenal among those working in the agricultural sector (35.3%). Of these variables, health decision-making (χ^2^ = 17.1; p < 0.001), age (χ^2^ = 184.9; p < 0.001), wealth (χ^2^** = **44.8; p < 0.001), religion (χ^2^ = 12.3; p < 0.01), education (χ^2^ = 52.9; p < 0.001), partner’s education (χ^2^ = 42.9; p < 0.001), frequency of reading newspaper (χ^2^ = 21.8; p < 0.001), frequency of watching television (χ^2^ = 13.0; p < 0.01) and occupation (χ^2^ = 85.2; p < 0.001) had significant associations with unmet need for contraception (see Table 1).

### Binary Logistic Regression on healthcare decision making capacity, socio-demographic variables and unmet need for contraception

Outcome of the binary logistic regression analysis has been reported in Table 2. Women who took their healthcare decisions alone were more likely to have unmet need for contraception [AOR = 1.35; CI = 1.08–1.70]. Compared with women aged 15–19, unmet need was nearly five times likely among women aged 45–49 [AOR = 4.58, CI = 3.05–6.86]. Richer women had lower odds of unmet need for contraception compared with the poorest [AOR = 0.77, CI = 0.61–0.97]. Women who were not affiliated to any religion were more likely to report unmet need compared with Muslim women [AOR = 1.53, CI = 1.07–2.19]. Compared with women who were not engaging in paid occupation, those into agriculture [AOR = 1.36, CI = 1.15–1.61] were more likely to have unmet need. Finally, those in the managerial sector had less odds of unmet need for contraception [AOR = 0.40, CI = 0.22–0.72] as indicated in Table 2.

## Discussion

Mali is one of the SSA countries with high fertility rate [29] and unmet need for contraception is a major public health problem [30]. In this study, we sought to test the hypothesis that women who take decisions alone on their healthcare are more likely to have no unmet need for contraception. We tested this by using the 2018 version of the MDHS data. After adjusting for confounders, the results showed that women who took their healthcare decision alone were more likely to have unmet need for contraception. The results in this current study on the association between healthcare decision making capacity and unmet need for contraception is different from previous studies on the association between increased household decision-making autonomy and unmet need for contraception [31–33].

Despite the inconsistency of our finding with previous studies, some possible pathways may account for the observation made in the current study. First, many women in a patriarchal society like Mali, have lesser decision-making power on major household issues due to economic dependence, lesser negotiation skills and cultural issues [31, 34]. Due to this, they might be able to take the decision alone, however, they may still need support such as financial assistance to be able to afford some of the direct and indirect costs associated with contraceptive uptake. Second, some studies have suggested that couples' joint participation in household decision making indicates a strong communication and cooperation [32, 35] and has the propensity to yield positive health outcomes. Invariably, when key decisions are taken jointly, partners share the responsibility of the decision. For example, Kabeer [36] found in Bangladesh that partners’ joint decisions taking in household has a strong positive association with health outcomes compared to sole/independent decisions. Again, evidence shows that a women’s autonomy paradigm is not fully functional in male dominated societies where cultural values place much emphasis on mutual dependence within families [37]. Within these societies, even the status of women’s autonomy are still culturally embedded in social relationships and strongly tied to the authority of men [38]. That notwithstanding, a qualitative study can help unearth the nuances surrounding healthcare decision making capacity and unmet need for contraception in Mali.

Aside the strong association between healthcare decision-making and unmet need for contraception, it is imperative to synthesize the results on the association between the covariates with previous evidence. It was found that women aged 45–49 had nearly five times higher odds of unmet need compared to women aged 15–19. This is inconsistent with what has been observed in several previous studies in other locations such as Pakistian [18], India [39], Eretria [33], Ethiopia [40, 41] and a review in low- and middle-income countries [42]. The probable reason for the high unmet need among those in the advanced ages might be that they have already attained their desired number of children and hence would like to use contraceptives to either limit or space [18]. Mali one of the countries with high fertility rate globally, and women with advanced age are likely to have more children compared with teenagers. The relatively older women are, therefore, more likely to have a strong need for contraception either for spacing or limiting, hence may have higher tendency of unmet need when their demand for contraception is not met for whatsoever reason.

Another important finding in our study was the association between wealth and unmet need for contraception. It was found that richer women had lower odds of unmet need compared with poorest women. The association is similar to previous studies in other low and middle income countries including Pakistan [18] and Zambia [43]. The explanation to this might be that wealthier women can have better access to modern contraceptives as compared to poorer women since the former will be able to afford both the direct and indirect costs associated with contraceptive use [18].

In terms of religious affiliation, it was found that women who were not affiliated to any religion were more likely to report unmet need compared with Muslim women. This finding suggests that Moslem women were less likely to have unmet need. Several studies have reported statistically significant association between religious affiliation and unmet need in India [44], Ethiopia [41], Bangladesh [45], Ghana [46] and a scoping review in low and middle income countries [26]. Various religious denominations have diverse discernments and self-beliefs and that can have either deleterious or positive influence on contraceptive use [47]. Members of Islamic religion and some Orthodox religions such as Catholics exhibit a strong opposition to contraceptive use [46]. By this, members of such religions may not use it in the first place.

It was also found that, compared with women who were not engaging in paid occupation, those into agriculture were more likely to have unmet need, whilst those in managerial sector had less likelihood of unmet need. This finding on the association between type of work and unmet need is similar to what was found in Sudan by Ali and Okud [14] as well as in Kenya by Nyauchi and Omedi [48]. It has similarly been reported from Ethiopia [49] and Saudi Arabia [50]. The possible explanation to this is the ability to pay and the ability to understand the use and importance associated with contraceptives.

### Strengths and limitations of the study

The major strength of this study is from the high quality of the data and large sample that was selected with utmost precaution to meet the inclusion/exclusion criteria for the purpose of the study. The analytical approach that was employed to analyse the data was also rigorous thereby strengthening the validity of our conclusion. The use of the nationally representative sample also allows generalization of the findings to similar women—15–49 years in Mali. This study also had some limitations that are worthy to be stated. The data was obtained through a cross-sectional study and so causations could not be established but only associations. The survey obtained retrospective information which was self-reported from participants spanning a 5-year period prior to the survey. Due to that the likelihood of recall bias cannot be ruled out in this study. The key independent variable (women healthcare decision-making capacity) and outcome variable (unmet need) including other covariates, were all self-reported by the women and were not verified by their partners. There is therefore, the possibility of social desirable responses. For example, a study by Ghuman, Lee and Smith [34] reported that wives consistently tend to underreport their participation in household decisions compared to their husbands’ report.

## Conclusion

In conclusion, we found that women who took their healthcare decision alone, women aged 45–49, women who were not affiliated to any religion and women in agriculture had higher odds of unmet need for contraception. On the other hand, richer women and women in managerial sector had lower odds of unmet need for contraception. To increase contraceptive usage and reduce high fertility rate in Mali, it is imperative to take these factors into consideration to strengthen existing policies geared towards fertility control and improvement in maternal health to achieve Sustainable Development Goal 3 and 5.

## Data Availability

The datasets supporting the conclusions of this article are available in the Measure DHS repository, https://dhsprogram.com/data/available-datasets.cfm.

## Abbreviations

AOR: Adjusted odds ratio
CI: Confidence interval
ES: Enumeration Sections
GCPH: General Census of Population and Housing
ICF: Inner City Fund
MDHS: Mali Demographic and Health Survey
OR: Odds ratio
PSU: Primary Survey Units
SSA: Sub-Saharan Africa
VIF: Variance inflation factor
WHO: World Health Organisation
